# How we treat primary immune thrombocytopenia in adults

**DOI:** 10.1186/s13045-023-01401-z

**Published:** 2023-01-19

**Authors:** Xin-guang Liu, Yu Hou, Ming Hou

**Affiliations:** 1 Department of Hematology, Qilu Hospital, Cheeloo College of Medicine, Shandong University, Jinan, China; 2 Shandong Provincial Key Laboratory of Immunohematology, Qilu Hospital, Cheeloo College of Medicine, Shandong University, Jinan, China

**Keywords:** Primary immune thrombocytopenia, Platelets, Diagnosis, Treatment

## Abstract

Primary immune thrombocytopenia (ITP) is an immune-mediated bleeding disorder characterized by decreased platelet counts and an increased risk of bleeding. Multiple humoral and cellular immune abnormalities result in accelerated platelet destruction and suppressed platelet production in ITP. The diagnosis remains a clinical exclusion of other causes of thrombocytopenia. Treatment is not required except for patients with active bleeding, severe thrombocytopenia, or cases in need of invasive procedures. Corticosteroids, intravenous immunoglobulin, and anti-RhD immunoglobulin are the classical initial treatments for newly diagnosed ITP in adults, but these agents generally cannot induce a long-term response in most patients. Subsequent treatments for patients who fail the initial therapy include thrombopoietic agents, rituximab, fostamatinib, splenectomy, and several older immunosuppressive agents. Other potential therapeutic agents, such as inhibitors of Bruton’s tyrosine kinase and neonatal Fc receptor, are currently under clinical evaluation. An optimized treatment strategy should aim at elevating the platelet counts to a safety level with minimal toxicity and improving patient health-related quality of life, and always needs to be tailored to the patients and disease phases. In this review, we address the concepts of adult ITP diagnosis and management and provide a comprehensive overview of current therapeutic strategies under general and specific situations.

## Introduction

Primary immune thrombocytopenia (ITP) is an acquired autoimmune bleeding disorder characterized by isolated thrombocytopenia (platelet count < 100 × 10^9^/L) in the absence of other etiologies. The incidence of the disease is approximately 2–10 per 100,000 adults each year, with a prevalence of 9–20 per 100,000 adults [1–3]. It is more common in females of childbearing age compared with males of the same age-group, and the incidence often reaches a peak in adults after 60 years of age with equal sex distribution. Many patients are asymptomatic or only present with mild mucocutaneous hemorrhage; however, severe bleeding occurs in 5–6% of patients [4]. A population-based study revealed a 1.3–2.2-fold increase in the mortality rate for adult ITP patients compared with the general population due to bleeding episodes, infection, and cardiovascular events [5]. Although the reported incidence of thrombosis was inconsistent, most studies demonstrated a slightly increased risk of thromboembolism in ITP patients [6–9]. Moreover, impaired health-related quality of life (HRQoL), including fatigue, has also been reported in adult patients with ITP [10].

The international working group (IWG) on ITP and the American Society of Hematology (ASH) both updated their guidelines for the diagnosis and management of ITP in 2019 [11, 12]. These landmark papers proposed a set of principles for optimizing the management of ITP and outlined several future research directions. In the past few years, there have been numerous advances in the understanding of ITP pathogenesis [13], which facilitate the development of new therapeutic agents targeting various underlying mechanisms. Indeed, great progress has been witnessed in optimizing treatments with novel agents or new combinations of already existing drugs. This review summarizes the current treatment strategies for adult ITP and discusses their applicability under general and specific situations.

## Pathogenesis of ITP

The decrease in peripheral blood platelet count of ITP patients results from accelerated platelet clearance and impaired platelet production. The loss of immune tolerance to platelet autoantigens is the critical upstream step in ITP pathophysiology, which leads to the abnormal recognition of platelet autoantigens and subsequent activation of T and B cells [14]. Autoantibody-mediated platelet destruction is the canonical mechanism of platelet destruction in ITP. Platelets coated by anti-glycoprotein (GP) autoantibodies are prematurely destroyed by macrophages via Fcγ receptors (FcγRs) in the reticuloendothelial system [15, 16]. Autoantibodies also accelerate platelet destruction through the induction of complement-dependent cytotoxicity (CDC) and platelet apoptosis [17–20]. Platelet autoantibody production involves complex interactions between macrophages/dendritic cells (DCs), T cells, and B cells. Defects in DCs, such as elevated surface CD86 expression, reduced indoleamine 2,3-dioxygenase (IDO) levels, and lowered numbers of plasmacytoid DCs, result in enhanced stimulation of autoreactive T cells and impaired induction of regulatory T cells (Tregs) in ITP [21–24]. Macrophages can process phagocytosed platelets and present autoantigens to T helper (Th) cells, which dictate the proliferation of B cells into autoantibody-secreting plasma cells, thus constituting a continuous autoantigen feedback loop [25, 26]. Decreased expression of the inhibitory FcγR on macrophages, enhanced M1 macrophage polarization, and increased number of inflammatory CD16^+^ monocytes have been observed in patients with active ITP and are related to the enhanced phagocytosis of opsonized platelets and autoreactive T-cell priming [15, 27, 28]. Moreover, a study showed that levels of CD86 and human leukocyte antigen (HLA)-DR were increased, but FcγR balance was not altered, on splenic macrophages from ITP patients [16]. CD4^+^ T cells provide the critical secondary signals to B cells for their activation and development into plasma cells. Dysregulated CD4^+^ T cell responses, including the broadly accepted dogma of excessive polarization toward the proinflammatory Th1, Th17, and Th22 lineages [29–31], resistance to activation-induced cell death (AICD) [32], and oligoclonal expansion of GP-specific T cells, are involved in the development of ITP [33]. Follicular Th (T_FH_) cell numbers are also increased in the periphery, spleen, and bone marrow of ITP patients, suggesting that their B cell help activity is upregulated [34, 35]. By contrast, circulating and splenic Tregs in ITP are found to be numerically decreased and functionally impaired, contributing greatly to the perpetuation of ITP [36–39]. Aside from macrophages, DCs, and T cells, abnormalities of B cell subsets, such as defective regulatory B cells (Bregs), expansion of memory B cells, and long-lived plasma cells, also play crucial roles in autoantibody production in ITP [40–43].

Approximately 20–40% of ITP patients do not have detectable anti-GP autoantibodies, suggesting alternative mechanisms of platelet destruction. Many studies have demonstrated that CD8^+^ cytotoxic T lymphocytes (CTLs) from peripheral blood or spleen of ITP patients can directly lyse platelets or induce platelet apoptosis through granzyme B and perforin [44–47], although the target platelet MHC class I peptides recognized by CD8^+^ T cells have not yet been identified. Desialylation-mediated platelet phagocytosis through hepatocyte Ashwell–Morell receptors in the liver is another mechanism of FcγR-independent platelet eradication in ITP [48, 49]. CTLs and anti-GP autoantibodies can induce the desialylation of platelet surface glycans, leading to premature platelet clearance [49, 50]. Recent studies have suggested a modified model in which desialylated platelets are mainly cleared by liver Kupffer cells through the interaction of macrophage galactose lectin (MGL) or Kupffer cell receptor (KCR, also known as C-type lectin domain family 4, CLEC4F) with N-acetylgalactosamine (GalNAc), galactose, and fucose on platelets [51, 52]. The exact mechanism by which desialylated platelets are cleared in ITP still awaits further investigation.

**Fig. 1:**
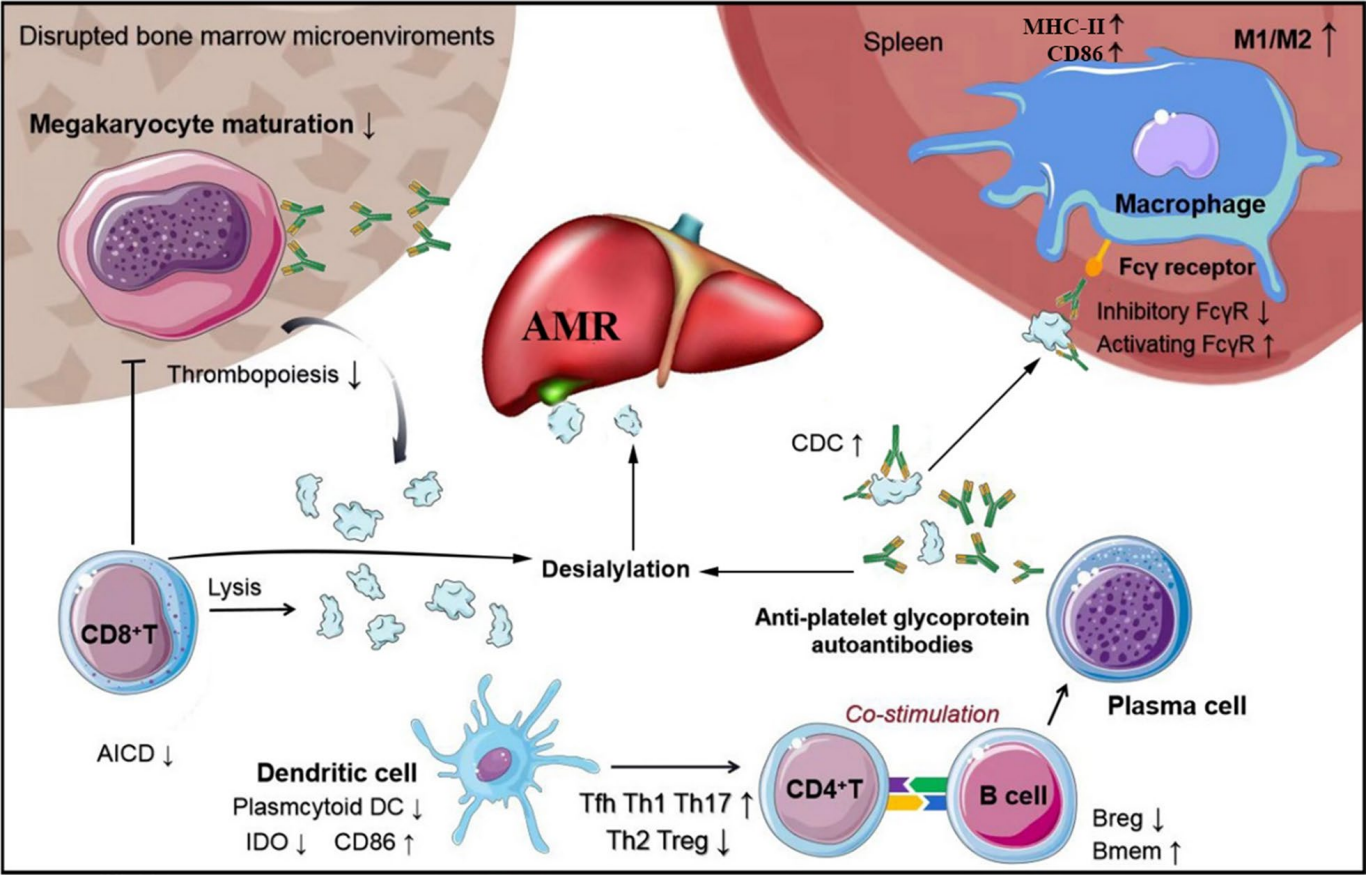
Pathophysiology of ITP. Thrombocytopenia in ITP is the result of both increased platelet destruction and suppressed platelet production. Platelet autoantigens are abnormally recognized, processed, and presented by DCs, and then CD4^+^ T helper cells are activated toward a proinflammatory profile, which dictate the differentiation of B cells into autoantibody-secreting plasma cells. Autoantibodies not only mediate platelet phagocytosis by macrophages through Fcγ receptors (FcγRs) but also induce platelet desialylation and subsequent clearance through hepatocyte Ashwell–Morell receptors (AMRs). Splenic macrophages have increased expression of major histocompatibility complex (MHC)-II and CD86 and can also present autoantigens to Th cells. CD8^+^ cytotoxic T lymphocytes (CTLs) can directly lyse platelets or induce platelet apoptosis. Moreover, autoantibodies and CTLs interfere with megakaryocyte maturation and apoptosis, leading to decreased platelet production in ITP. *AMR* Ashwell–Morell receptor, *FcγR* Fcγ receptor, *M1/M2* M1/M2 macrophage polarization, *CDC* complement-dependent cytotoxicity, *AICD* activation-induced cell death, *DC* dendritic cell, *IDO* indoleamine 2,3-dioxygenase, *Tfh* follicular T helper cell, *Th* T helper cell, *Treg* regulatory T cell, *Breg* regulatory B cell, *Bmem* memory B cell, *MHC-II* major histocompatibility complex-II, ↓ means decreased or downregulated, ↑ means increased or upregulated

Platelet turnover studies have revealed that thrombopoiesis is decreased or normal in most ITP patients, and the absolute reticulated platelet counts are reduced [53–55], suggesting relatively insufficient platelet production in the bone marrow. After binding to megakaryocytes, anti-GP autoantibodies can interfere with the differentiation, maturation, and apoptosis of megakaryocytes [56–58]. Platelet autoreactive CTLs in bone marrow can also impair megakaryocyte apoptosis through the downregulation of Fas and upregulation of Bcl-xl [47, 59]. Recruitment of CTLs into bone marrow was found to be elevated in ITP patients due to increased expression of VLA-4 and CX3CR1 [60]. Additionally, the abnormal bone marrow microenvironment as demonstrated by defects in mesenchymal stem cells and endothelial progenitor cells [61–63], dysregulated T and B cell subsets [40, 64], and relatively insufficient thrombopoietin levels are all related to impaired platelet production in ITP [65]. The hypothesized pathophysiology of ITP is summarized in Fig**. ****1**. Of note, the above-mentioned pathological mechanisms may play varying roles in different patients due to the heterogeneity of the disease. Identifying which mechanism is dominant in each case is critical for the implementation of pathogenesis-oriented individualized treatment.

## Diagnosis and prognosis of ITP

ITP remains a diagnosis of exclusion due to the lack of a “gold standard” diagnostic test. History taking, physical examination, complete blood count, and peripheral blood film assessment are the basic workups for suspected patients. Splenomegaly and constitutional symptoms (e.g., fever, weight loss, or lymphadenopathy) are unusual and, if present, should be investigated further to exclude the presence of other underlying diseases. Several additional tests, including reticulocyte counts, quantitative immunoglobulin (Ig) levels, blood groups, and serologic screening for HCV/HBV/HIV, are also recommended as the basic evaluation by the updated international consensus report [11]. Therefore, the diagnosis of ITP can be established with careful history taking and physical examination, as well as a review of peripheral blood smears and minimal further testing in most patients. However, the application of this diagnostic criterion will lead to misdiagnosis in a small proportion of patients with suspected ITP. According to several population-based studies, myelodysplastic syndrome (MDS) and secondary ITP, such as thrombocytopenias due to systemic autoimmune diseases, malignancies, primary immune deficiency, or drug exposure, accounted for approximately 20% of the misdiagnosis [3, 66, 67]. To minimize the misdiagnosis rate, the Chinese ITP guidelines also recommend bone marrow examination and tests for antinuclear antibodies (ANAs), anti-phospholipid antibodies (APLAs), anti-thyroid antibodies, thyroid function, and coagulation parameters (prothrombin time, activated partial thromboplastin time, fibrinogen level, and D-dimer) as the basic evaluation [68, 69]. Bone marrow examination includes aspiration smear, biopsy, flow cytometry, and cytogenetic analysis. These differences may reflect a higher percentage of alternative diagnoses in China, as the incidence of aplastic anemia (AA) is higher and the average age of onset of MDS is lower than that in Western countries [70–72]. Therefore, bone marrow examination can help reduce the misdiagnosis rate and shorten the time to diagnosis. The Chinese ITP guidelines also list specialized laboratory assays that include the detection of anti-GP autoantibodies by monoclonal antibody-specific immobilization of platelet antigen (MAIPA) [73] or flow cytometric immunobead array, serum thrombopoietin levels, *Helicobacter pylori* infection, direct antiglobulin test (DAT), and the quantification of parvovirus, Epstein–Barr virus, and cytomegalovirus deoxyribonucleic acid (DNA). These assays do not need to be routinely performed for ITP diagnosis; however, targeted testing may be useful in certain cases with special symptoms, signs, and laboratory findings. Anti-GP autoantibody detection is highly specific for ITP diagnosis, while the sensitivity is relatively low. The presence of anti-GP autoantibodies is helpful to confirm the diagnosis of immune thrombocytopenia, while it cannot distinguish the primary from the secondary ITP [74, 75]. Serum TPO levels are normal or slightly increased in ITP patients, while remarkably elevated in patients with AA or hypoproliferative MDS [65, 76], thus being potentially valuable for the differential diagnosis of ITP. Detection of *Helicobacter pylori* infection using the urea breath test or stool antigen test can be performed in patients with dyspeptic symptoms, especially in areas with a high prevalence of *Helicobacter pylori* infection. DAT is suitable for ITP patients with clinical or laboratory evidence of hemolysis. Because of the substantial reduction in the cost of next-generation sequencing, genomic assays, such as whole-exome sequencing (WES), whole-genome sequencing, or targeted gene sequencing, may provide valuable information for multirefractory patients or cases who will undergo splenectomy to exclude MDS, bone marrow failure syndrome, and inherited thrombocytopenias [77, 78]. Laboratory tests in the differential diagnosis of ITP are summarized in Table**1**.

**Table 1:** Differential diagnosis of ITP and suggested tests to exclude the alternative causes of thrombocytopenia

Alternative diagnosis	Clinical characteristics	Laboratory tests
Pseudothrombocytopenia	No thrombocytopenia-related symptoms (in vitro phenomena)	Platelet clumping on blood smear due to EDTA-dependent agglutinins
Acute or chronic Infections (HBV/HCV/HIV/EBV/CMV/B19/Zika/*H. pylori*)	High-risk populations History, suggestive symptoms or signs	Serologic and PCR tests for viral infection Urea breath test for *H. pylori*
Drug-induced (heparin, quinine, antibiotic, NSAIDs, etc.)	History of drug exposure	Drug-dependent antibody tests
Vaccine-associated	Recent history of vaccination (< 6 weeks)	–
Connective tissue diseases (SLE, rheumatoid arthritis, anti-phospholipid syndrome, etc.)	Fever, rash, arthralgias, mouth ulcers, hair loss, abortions, and thromboembolism	Targeted serologic tests (ANAs, anti-dsDNA antibodies, anti-CCP antibodies, APLAs, etc.)
Lymphoproliferative disorders (CLL, Hodgkin’s lymphoma, etc.)	Fever, weight loss, night sweats, lymphadenopathy, splenomegaly	Lymph node biopsy, bone marrow examination, imaging examination
Immunodeficiency syndrome (common variable immunodeficiency)	Young age, recurrent infections, colitis, lymphadenopathy	Ig levels, lymphocyte ph, genetic testing
Evans syndrome	Hemolysis and thrombocytopenia	Coombs test
Bone marrow malignancy (MDS, leukemia, etc.)	Suggestive symptoms or signs (fever, splenomegaly, bleeding, etc.)	Blood smear, bone marrow examination
AA	Pancytopenia	Blood smear, bone marrow examination
Thrombotic microangiopathy (thrombotic thrombocytopenic purpura, hemolytic uremic syndrome)	Hemolysis, neurologic symptom, fever, renal damage	Blood smear (schistocytes), haptoglobin, LDH, ADAMTS13 level
DIC	Precipitating events, severe patients, multiple organ damages	Coagulation tests, blood smear
Inherited or congenital diseases (Wiskott–Aldrich syndrome, Bernard–Soulier syndrome, MYH9-related disease, type IIb vWD, etc.)	Young-onset thrombocytopenia, family history, congenital abnormalities (deafness, cataract, development delay)	Blood smear, genetic testing

*EDTA* ethylenediaminetetraacetic acid, *NSAID* nonsteroidal anti-inflammatory drug, *SLE* systemic lupus erythematosus, *ANA* antinuclear antibody, *APLA* anti-phospholipid antibody, *CLL* chronic lymphocytic leukemia, *MDS* myelodysplastic syndrome, *AA* aplastic anemia, *LDH* lactate dehydrogenase, *DIC* disseminated intravascular coagulopathy *ph* phenotype

It should be emphasized that the response to treatments, especially intravenous immunoglobulin (IVIg), is of great value for the confirmation of ITP diagnosis. As an immune-mediated disorder, a large proportion of patients with ITP will undergo repeated outbreaks and tend to run a protracted course, or are eventually diagnosed with thrombocytopenia secondary to connective tissue diseases such as systemic lupus erythematosus (SLE). Therefore, it is critical to re-evaluate the diagnosis during the course of the disease, especially in patients who respond poorly to treatment and cases with new symptoms, signs, and new laboratory findings.

According to the disease duration, ITP can be divided into newly diagnosed (< 3 months), persistent (3–12 months), and chronic (> 12 months) phases. Adult patients are more likely to develop chronic disease than children. Moreover, higher platelet counts at diagnosis and the presence of ANAs and anti-thyroid antibodies may serve as predictors of disease chronicity [79–81]. Severe ITP refers to patients with clinically relevant bleeding of sufficient magnitude to mandate disease-directed treatments, or cases showing new bleeding symptoms requiring additional interventions or an increase in drug dose [69, 82]. The IWG does not use the term “refractory” anymore because of the declining rate of splenectomy [11]. However, the description of “refractory ITP” is often reserved for patients who have failed multiple lines of therapy, especially thrombopoietic agents and rituximab, or splenectomy [67, 69, 83].

Bleeding severity in ITP patients may be graded using the well-established ITP-specific bleeding assessment tool (ITP-BAT), as it is one of the basic determinants of treatment initiation and response evaluation [84]. Identification of patients at high risk of severe bleeding will help start the treatment timely to prevent fatal hemorrhage, even in those with higher platelet counts, whereas this remains a big challenge. Older age, low platelet counts, the presence of comorbidities, and concurrent anticoagulants or antiplatelet medications contribute to an increased risk of bleeding [85–87]. Data from the CARMEN-France registry indicated that anticoagulant exposure was the major risk factor for severe bleeding in very elderly ITP patients aged ≥ 80 years, and thus for whom more close monitoring was needed [88]. Disease course also impacts bleeding manifestations, and the rate of bleeding-related events in newly diagnosed patients is significantly higher than in chronic cases [89]. Intracranial hemorrhage (ICH) occurs in 1.5–1.8% of adult ITP patients [86]. This proportion of patients often shows more severe thrombocytopenia and bleeding symptoms, such as hematuria and visceral hemorrhage, and usually have a history of head trauma. A Chinese nationwide multicenter study reported that the mortality rate of ICH in adult ITP was 33.8%, and the risk factors of mortality included intraparenchymal hemorrhage, platelet counts ≤ 10 × 10^9^/L, serious infections, severe preceding bleeding events, and Glasgow coma scale [90]. Actually, occult cerebral microbleeds (CMBs) occur in almost 50% of ITP patients with platelet counts < 30 × 10^9^/L, and the occurrence is associated with lower nadir platelet counts, longer disease duration, and higher organ bleeding scores [91]. Platelet function and platelet microparticles also influence bleeding severity in ITP [92–94], but their exact roles remain to be evaluated.

## Treatment of ITP

The main goals of ITP treatment are to prevent bleeding and maintain the platelet count above a safe level to minimize the bleeding tendency. Notably, the safe lower limits for platelet counts vary among patients and are related to individualized bleeding risks. The updated ASH guidelines recommend the initiation of disease-specific treatments if the platelet counts are < 30 × 10^9^/L [12], while it is a recommendation based on very low certainty in the evidence of effects. The association between platelet count and bleeding events is poor in ITP patients with platelet counts > 10 × 10^9^/L. This is exactly the case even in very elderly patients, and the bleeding severity of whom was found to be strongly associated with anticoagulant exposure [88]. Bleeding episodes are more influential than platelet counts in the clinical decision-making process of treatment initiation. Patient expectations and preferences, costs and availability of treatments, and factors that influence bleeding risks, such as age, comorbidities, concurrent therapies, lifestyle, and profession, should also be considered. In addition, HRQoL is an important factor that needs to be taken into account when making treatment plans. It is noteworthy that medications used in ITP, such as corticosteroids and immunosuppressants, may sometimes have detrimental effects on patient health status, resulting in a poor overall quality of life perhaps worse than the disease itself. Therefore, an optimized treatment strategy should improve platelet recovery with minimum toxicity and elevated HRQoL, and should always be tailored to the patients and disease phases.

**Fig. 2:**
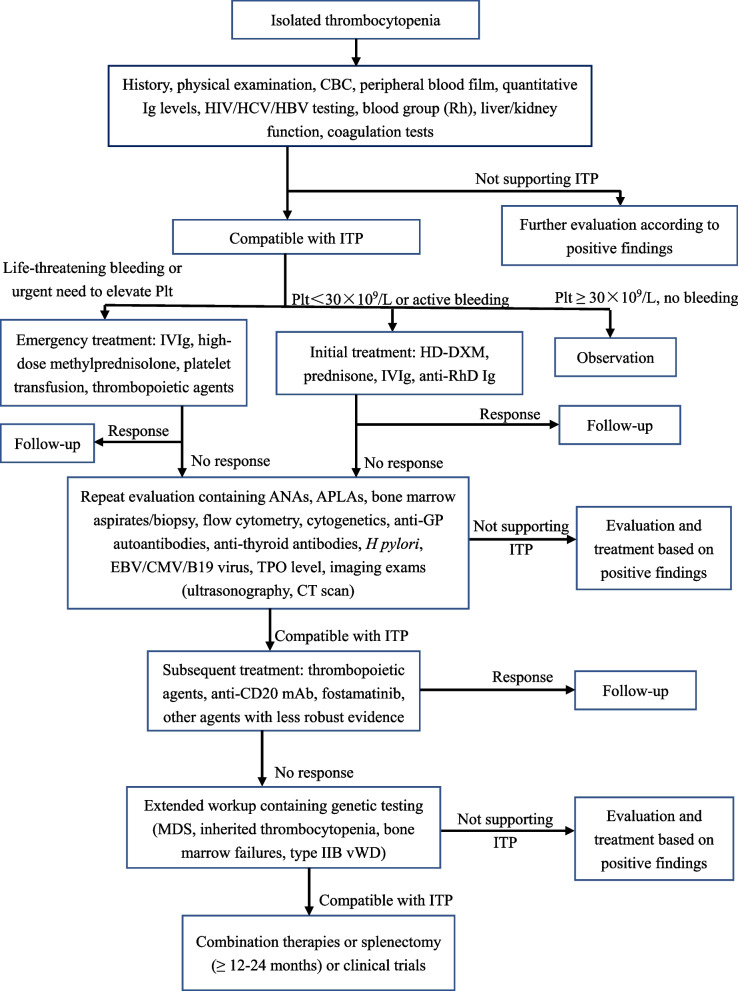
Overview of diagnosis and treatment of adult ITP. *CBC* complete blood count, *Plt* platelet count, *IVIg* intravenous immunoglobulin, *HD-DXM* high-dose dexamethasone, *ANAs* antinuclear antibodies, *APLAs* anti-phospholipid antibodies, *TPO* thrombopoietin, *CT* computerized tomography, *MDS* myelodysplastic syndrome, *vWD* von Willebrand disease

According to the updated international consensus report, ITP treatment can be broadly divided into initial/emergency treatment, subsequent treatment, and regimens for patients who fail multiple treatments [11]. The diagnosis and management procedures are summarized in Fig. 2. New therapeutic agents and emerging evidence have facilitated a gradual shift in ITP therapeutic models away from immune suppression, especially for persistent and chronic patients.

## Initial treatment

Corticosteroids remain the initial cornerstone therapy for ITP patients without relative contraindications. Pulsed high-dose dexamethasone (HD-DXM, 40 mg/day for 4 days, a maximum of 3 cycles) or predniso(lo)ne (1 mg/kg/day for 2 weeks, not exceeding 80 mg/day, with a gradual taper and final stop by 6–8 weeks) is the most commonly used regimen, with an initial response rate of 60–80%. Approximately 20–40% of the patients could maintain the response after corticosteroid discontinuation [95, 96]. Prolonged corticosteroid courses are not recommended due to the adverse effects. Although sporadic studies have reported that HD-DXM could induce higher sustained response rates (SRR) than prednisone [97, 98], most randomized clinical trials (RCTs) and a well-conducted meta-analysis did not find any difference in SRR between HD-DXM and prednisone at 6 months or longer [99, 100]. It is notable that HD-DXM could induce a more rapid response and seems to have a better safety profile (lower incidence of steroid-related comorbidities) than prednisone [99]; however, caution needs to be taken when interpreting the safety data as the prolonged use of prednisone (≥ 8 weeks) in that RCT. Nevertheless, HD-DXM might lead to the occurrence of neuropsychiatric complications in very elderly patients and those with a history of psychiatric disease [101] and should be avoided. Under such situations, a shorter duration (e.g., 4 weeks) of prednisone and faster tapering are more favored.

IVIg is another commonly used initial therapy that can raise the platelet counts rapidly in more than 80% of newly diagnosed ITP patients; however, it is relatively expensive and the response is usually transient [102]. A single-arm observational study suggested that bleeding severity, rather than platelet count, could serve as a relevant indicator for IVIg treatment [103], while more data are needed to validate the finding. IVIg is usually administered at a dose of 1 g/kg/day for 1–2 days or 0.4 g/kg/day for 5 days with similar efficacy [11, 102]. A prospective case–control study showed that IVIg at a lower dosage of 0.2 or 0.3 g/kg/day had a comparable response rate and onset time to the conventional dosage of 0.4 g/kg/day [104], suggesting the possibility of treating ITP patients more cost-effectively by lowering the IVIg dosages. It is notable that IVIg is a blood product that is sometimes in short supply and therefore should be used sparingly. The decision on IVIg treatment initiation ought to depend mainly on the bleeding severity grade rather than the platelet count. In addition, IVIg should be used with caution in patients with impaired renal function and cases with increased thrombosis risks. The presence of anti-GPIb/IX autoantibodies might serve as a predictor of poor response to IVIg [105], but this finding was inconsistent among several reports and still needs to be confirmed [74, 106].

Intravenous anti-RhD Ig has been proposed as an alternative to IVIg in Rh-positive patients with intact spleens. Anti-RhD-coated erythrocytes can saturate macrophage FcγRs and inhibit autoantibody-mediated platelet destruction. A single infusion of anti-RhD Ig at a dose of 50 μg/kg could lead to an overall response rate of 65% in newly diagnosed ITP patients, with a median response duration of 3–4 weeks [107]. The major concern regarding anti-RhD Ig therapy is the occurrence of rare but life-threatening episodes of severe intravascular hemolysis with acute renal failure and disseminated intravascular coagulation (DIC). Anti-RhD Ig is not widely used because it is not available in many countries.

Neither corticosteroids, IVIg, nor anti-RhD Ig is able to modify the natural course of ITP by preventing its chronic evolution. Hence, improving the initial response and maintaining the sustained response remain a big challenge. Under such circumstances, early combination strategies to intensify the effect of initial therapy have been developed. Two large RCTs investigated the upfront use of HD-DXM plus rituximab in treatment-naïve ITP patients. The results showed that the addition of rituximab to HD-DXM did not elevate the early response rates, while significantly improved the SRR at 6 and 12 months. However, an extended follow-up revealed no overall response benefit of rituximab inclusion beyond 1 year [108, 109]. A meta-analysis also showed that the combination treatment yielded a similar improvement in SRR within 1 year but no superiority in relapse rate over time [110]. Taking into account the added toxicity and costs, the addition of rituximab to corticosteroids is not routinely recommended for newly diagnosed ITP patients according to the current guidelines, and more robust data are needed to assess this combination regimen.

In contrast to the conventional treatments that function mainly by decreasing platelet destruction, thrombopoietic agents functioning by stimulating platelet production have significantly changed the management of ITP. Due to their non-immunosuppressive character, thrombopoietic agents are often used off-label for newly diagnosed ITP patients in real-world settings. Several single-arm studies reported that HD-DXM in combination with eltrombopag had favorable SRR at 6 months (56.5–75%) and good tolerability in newly diagnosed treatment-naïve ITP patients [111, 112]. In addition, more intensified strategies using the combination of eltrombopag or romiplostim, low-dose rituximab, and HD-DXM have been proposed and tested in small pilot studies [113, 114]; however, the improvement in relapse-free survival yielded by the three-drug combination still needs validation. In a large prospective RCT, we recently showed that HD-DXM plus a limited course of recombinant human thrombopoietin (rhTPO) elicited a higher response rate at day 14 and month 6, and a slightly higher treatment-free remission rate thereafter than HD-DXM monotherapy [115]. The efficacy and safety of the upfront use of thrombopoietin receptor agonists (TPO-RAs) in combination with corticosteroids are still being evaluated in several ongoing RCTs.

Other combination regimens that have been tested in RCTs for the initial management of ITP include mycophenolate mofetil (MMF) plus corticosteroids, all-trans retinoic acid (ATRA) plus HD-DXM, and oseltamivir plus HD-DXM. The open-label FLIGHT trial showed that MMF up to 1 g twice daily plus corticosteroids elicited a significantly higher complete response (CR) rate (91.5% vs. 63.9%) and fewer treatment failures (22% vs. 44%) than corticosteroid monotherapy. There was no difference in bleeding, rescue treatments, and severe adverse events between the 2 arms. However, MMF had an obvious detrimental effect on patient HRQoL [116]. ATRA is an active derivative of vitamin A that has multiple immunomodulatory activities. Huang et al. observed in a phase II RCT that ATRA (10 mg twice daily for 12 weeks) plus HD-DXM induced a significantly higher SRR at 6 months than HD-DXM monotherapy (68% vs. 41%) in newly diagnosed ITP patients, and this novel combination treatment did not increase the occurrence of severe adverse events [117]. With the recognition of desialylation-mediated platelet clearance in ITP pathogenesis, oseltamivir, a widely used anti-influenza sialidase inhibitor, was found to be the potential in ameliorating thrombocytopenia [118]. Along similar lines, we conducted a multicenter RCT to assess the combination of oseltamivir (75 mg twice daily for 10 days) with HD-DXM versus HD-DXM alone as the initial treatment for ITP. It was encouraging that patients in the combination arm achieved a higher initial response rate (86% vs. 66%) and SRR at 6 months (53% vs. 30%) than those in the HD-DXM monotherapy arm; however, the superiority of response was gradually lost, and there was no statistical significance in SRR between the 2 arms after 1 year [119]. The wide availability and affordability of ATRA and oseltamivir warrant further evaluation of these initial combination treatments in late-stage RCTs.

## Emergency treatment

In patients with life-threatening bleeding or those requiring emergency surgery, combination treatments are often needed to increase the platelet count rapidly. So far, there is still a lack of large RCT comparing the emergency treatment of ITP, and most recommendations are still based on observational studies or expert opinions. The IWG recommends the concomitant use of IVIg, intravenous corticosteroids, and platelet transfusion with general supportive care. TPO-RAs can also be administered concurrently at relatively more aggressive dosages to increase the response rate and reduce early relapse [11], especially for patients with refractory ITP in an emergency bleeding situation [120]. Vinca alkaloids may also be considered, but the risk of peripheral neuropathy should be noted. Other agents that do not target ITP pathogenesis, such as antifibrinolytics, recombinant activated factor VII, and oral contraceptives (for female patients with menorrhagia), can be helpful in stopping the active bleeding under certain conditions.

## Subsequent treatment

Although newly diagnosed ITP patients have relatively high response rates to initial treatments, the vast majority of them undergo disease relapse, and about 60–70% of the patients will progress to persistent or chronic ITP [121]. Subsequent treatments in patients who fail the initial therapy can be classified into medical and surgical treatments, and therapeutic choices are primarily based on resource availability, required onset time, side effects, and patient or physician preference. This is due to the absence of RCTs directly comparing these subsequent treatment options and the lack of biomarkers to guide treatment decisions. Subsequent medical options with robust evidence include thrombopoietic agents, rituximab, and fostamatinib. We encourage eligible patients to participate in well-designed clinical trials. When choosing the subsequent therapy, patients should be informed of the efficacies and limitations of the different options, and are encouraged to participate in treatment decision-making.

### Thrombopoietic agents

TPO-RAs have dramatically changed the treatment modality of ITP, making the avoidance of immunosuppressive agents possible in disease management. The Food and Drug Administration (FDA) and European Medicines Agency (EMA) have approved 3 TPO-RAs for the management of persistent or chronic ITP: eltrombopag, romiplostim, and avatrombopag. Additionally, rhTPO and hetrombopag are also licensed as second-line treatments for ITP in China. The reported overall response rates of these above-mentioned thrombopoietic agents range from 70 to 90% in RCTs for previously treated chronic patients, and 50–60% of them could maintain the response with prolonged treatment duration [122]. Several studies suggested that patients with a remarkable elevation in the baseline TPO level responded poorer than those with a relatively normal TPO level [123, 124], whereas the cutoff value for TPO remains to be elucidated. A systematic review including 13 RCTs indicated that eltrombopag and romiplostim were associated with significantly higher treatment response (risk ratio [RR] = 2.77) and durable response rates (RR = 7.52), lower incidence of bleeding events (RR = 0.8), and a decreased proportion of patients needing rescue therapy (RR = 0.5) compared with placebo [125]. A more recent meta-analysis including more RCTs also revealed that TPO-RAs had significantly lower treatment failure (RR = 0.42) and all-cause mortality rates (RR = 0.21) than the placebo, and TPO-RAs did not increase the occurrence of adverse events [122]. Mei et al. recently showed in an RCT that rhTPO (300 U/kg/day for 2 weeks) could induce a more rapid response and higher initial response rate within 15 days in persistent ITP patients compared with eltrombopag (25 mg/day for 2 weeks); however, the observation period was too short to draw further conclusions [126]. Apart from that study, no RCT has compared the efficacy and safety of thrombopoietic agents head-to-head. A network meta-analysis comparing eltrombopag, romiplostim, and rhTPO plus rituximab in persistent ITP showed the superiority of romiplostim and eltrombopag over rhTPO plus rituximab in platelet response and treatment safety [127], though attention should be paid when interpreting the conclusions owing to the heterogeneity of the studies included.

Eltrombopag, avatrombopag, and hetrombopag are small-molecule non-peptide TPO-RAs with similar efficacies of approximately 70–80% in persistent or chronic patients. Eltrombopag is generally initiated orally at a dose of 50 mg/day (25 mg/day in patients of East Asian descent), which is titrated between 12.5 and 75 mg/day to keep the goal platelet count of 50–200 × 10^9^/L. Similarly, hetrombopag is typically started at a dose of 2.5 mg/d, which is adjusted between 2.5 and 7.5 mg/day to maintain the goal platelet counts. Avatrombopag is administered at an initial dose of 20 mg/day and can be titrated to a maximum dose of 40 mg/day in treating ITP [128]. Both eltrombopag and hetrombopag interact with food because of their metal ion-chelating ability. By contrast, avatrombopag has no food–drug interactions and is better absorbed with food.

Romiplostim is a peptide with 4 TPO receptor binding sites linked to an IgG1-Fc component. It is injected subcutaneously at a starting dose of 1 μg/kg/week, which can be increased to a maximum of 10 μg/kg/week to reach the goal platelet counts. An aggressive starting dose of 3 μg/kg/week is also acceptable, as the majority of the patients require doses ≥ 3 μg/kg/week to respond [129]. rhTPO is a glycosylated full-length TPO only available in a few countries. It is administered subcutaneously at a dose of 300 U/kg/day for 2 weeks, with a response rate of 60–70% in corticosteroid-resistant or relapsed ITP patients [130]. Maintenance therapy with rhTPO could keep 85% of ITP patients in remission at a 3-month follow-up [131]. The selection of these thrombopoietic agents mainly depends on drug availability, patient-preferred administration form, and anticipated adherence. Furthermore, failing one thrombopoietic agent does not preclude the use of another, as there is accumulating evidence supporting TPO-RA agent switching due to treatment failure, which could induce a platelet response in up to 50–75% of patients [132–134].

Although TPO-RAs are not regarded as curative agents, somewhat unexpectedly, approximately 10–50% of patients after extended TPO-RA exposure are able to taper and eventually discontinue treatment without relapse [135–137]. Nevertheless, predictors of long-term response after TPO-RA discontinuation are lacking, but progressive tapering may be preferentially attempted in patients with a stable CR [137]. In an expert consensus based on the RAND/UCLA modified Delphi method, TPO-RAs can be tapered in patients with normal or above normal platelet counts, no history of major bleeding, and no requirement for an intensification of treatment in the past 6 months; meanwhile, duration of ITP and TPO-RA taking as well as time to response (TTR) after treatment does not affect the decision on TPO-RA tapering [138]. As for how to taper TPO-RAs, the first approach is decreasing the dose periodically to the minimum available dose but maintaining the time interval between doses. Eltrombopag, avatrombopag, and hetrombopag can also be tapered by maintaining the dose but periodically prolonging the time interval between doses. Our anecdotal experience in tapering oral TPO-RAs involves increasing the time interval preferentially in patients who received a low starting dose, while decreasing the dose preferentially in cases with a median/high initiating dose.

Thrombopoietic agents are generally well tolerated. Thrombotic risk remains a major concern, though its elevation has not been reported in numerous RCTs [139]. Uncontrolled observational studies indeed have demonstrated a twofold–threefold increase in the occurrence rate of thrombotic events in TPO-RA-treated patients [140]; thus, extra attention is needed, especially in patients at a high risk of thromboembolic events. Fewer than 10% of patients with prolonged TPO-RA exposure might show a moderate increase in bone marrow reticulin deposition, which was reversible upon medication withdrawal. Liver function should be monitored regularly in patients treated with eltrombopag or hetrombopag due to the potential hepatotoxicity. Other commonly observed side effects of these agents include headache, arthralgia, myalgia, and dizziness, which usually do not require additional treatment.

### Rituximab

Rituximab is a chimeric monoclonal antibody targeting CD20-expressing B cells. It has been commonly used as an off-label treatment for ITP for nearly 2 decades. The short-term response rates range from 60 to 70% in patients treated with the standard 4 weekly doses of 375 mg/m^2^ rituximab, and the response is usually achieved within 4–8 weeks [141]. Alternative dosing schedules of rituximab, such as 100 mg/week for 4 weeks, 1000 mg on day 1 and day 15, or a single dose of 375 mg/m^2^, have been explored in ITP patients showing similar short-term efficacy [142]. With the clearance of rituximab from the body and the gradual recovery of B cells, most patients will relapse after 6 months. According to a pooled analysis of several observational studies, SRRs at 1, 2, and 5 years were 38%, 31%, and 21%, respectively [143]. In spite of the high relapse rate, most relapsed patients still responded to retreatment with rituximab. Several studies have suggested that young female patients with a relatively short disease duration often respond better than patients of other demographics [141, 143]. Other proposed predictive factors, such as anti-GP autoantibodies and ANAs [144, 145], still require validation. Acute infusion reactions are common and can be handled easily, whereas the major concerns regarding rituximab include an increased risk of infection due to chronic B cell depletion, hypogammaglobulinemia, late-onset neutropenia, and the extremely rare progressive multifocal leukoencephalopathy. Importantly, rituximab is associated with an increased risk of HBV reactivation and is thus unsuitable for patients with evidence of active HBV infection [11, 69]. However, in certain chronic HBV-infected cases who do not have other effective therapeutic options for ITP, rituximab could be given with concomitant antiviral treatments and close monitoring of HBV DNA load and disease flare. In addition, rituximab can blunt the immune responses to different vaccines, including COVID-19 vaccines; therefore, special attention should be paid to rituximab use during the COVID-19 pandemic.

### Fostamatinib

Fostamatinib is an oral spleen tyrosine kinase (Syk) inhibitor approved for ITP patients who have failed at least one previous regimen. It blocks the downstream signal transduction initiated by activating FcγRs and B cell receptors (BCRs), leading to the amelioration of autoantibody-mediated platelet phagocytosis. The starting dose of fostamatinib is 100 mg twice daily, which can be uptitrated to 150 mg twice daily in patients with an inadequate response. In a phase III RCT of previously heavily treated patients with persistent/chronic ITP, the overall response (platelet counts ≥ 50 × 10^9^/L within 12 weeks of treatment) rate was 43% for fostamatinib-treated patients, and the median TTR was 15 days. Eighteen percent of fostamatinib-treated patients achieved a stable response (platelet counts ≥ 50 × 10^9^/L on at least 4 of 6 biweekly visits during weeks 14 to 24) [146]. An extended follow-up study demonstrated a durable response rate of 44% in patients on fostamatinib maintenance therapy for a median of ≥ 28 months [147]. The most commonly observed adverse events include diarrhea, hypertension, nausea, and transaminase elevation, which are generally mild to moderate and can be observed in up to 30% of patients.

### Splenectomy

Splenectomy remains the most effective therapy for corticosteroid-resistant or relapsed ITP patients by removing the major site of platelet phagocytosis and autoantibody production. Although splenectomy is becoming less preferred nowadays due to the availability of emerging non-surgical medications, it still offers the best chance for long-lasting remission, with an estimated durable response rate of 60–70% [148], even in TPO-RA and/or rituximab-resistant or relapsed patients [149]. There is also evidence that even though CR was not achieved after splenectomy, most patients displayed a milder course of the disease and responded better to medical treatment [149, 150]. Most guidelines recommend deferring splenectomy for 12–24 months after diagnosis as some patients have a chance of spontaneous remission or stabilization of platelet count at a hemostatic level [11, 12]. Reconfirmation of the disease diagnosis is necessary before splenectomy, and under such circumstances, tests such as bone marrow evaluation, assays of anti-GP autoantibodies, and serum TPO levels are helpful. Laparoscopic splenectomy is as effective as open splenectomy for alleviating patient’s thrombocytopenia, but it has lowered perioperative risks, shortened hospitalization, and decreased blood loss, thus becoming the standard procedure in most centers. To date, there is still a lack of reliable predictors of response to splenectomy. Autologous platelet scanning is capable of determining the predominant site of platelet sequestration, though not yet widely available.

Splenectomy is associated with a persistent 3–fourfold increase in the risk of venous thromboembolism (VTE) among ITP patients [74, 148, 151], and the risk remains elevated both early (< 90 days) and late (≥ 90 days) after splenectomy [152]; therefore, postoperative thromboprophylaxis is appropriate in high-risk cases. There is also an increased risk of infection in splenectomized ITP patients, among whom the reported incidence of sepsis ranges from 2.1 to 6.0% [151, 153]. The necessity for antibiotic prophylaxis in splenectomized adult patients is still undetermined, and we suggest antibiotic prophylaxis in high-risk patients, such as immunocompromised cases, or those with a poor response to vaccination. Moreover, patients should be treated empirically with antibiotics at the first sign of infection. Vaccinations against encapsulated organisms (*Streptococcus pneumoniae*, *Neisseria meningitidis*, and *Haemophilus influenzae*) should be given ≥ 2 weeks before splenectomy and maintained. The incidence of surgical complications and mortality rates are relatively higher in patients > 60 years of age. Therefore, splenectomy should be considered a last resort and only be performed after a comprehensive assessment of the disease in elderly patients.

### Novel treatments currently under investigation

Bruton’s tyrosine kinase (BTK) has emerged as a promising target for autoimmune disorders owing to its critical role in transmitting signals originating from FcγRs and BCRs. Rilzabrutinib is an oral, reversible, covalent small-molecule BTK inhibitor currently being tested for ITP management in late-stage clinical trials. In a recently published phase I–II clinical trial, 40% of previously treated ITP patients achieved response (platelet counts ≥ 50 × 10^9^/L) after rilzabrutinib administration (200 mg once daily to 400 mg twice daily), with a median TTR of 11.5 days. All tested doses of rilzabrutinib were well tolerated, with no or only mild adverse effects. Responded patients could keep the platelet count ≥ 50 × 10^9^/L throughout 65% of the study weeks [154].

The neonatal Fc receptor (FcRn) extends the half-life of IgG molecules by reducing their intracellular degradation in vascular endothelial cells. FcRn blockade interrupts IgG recycling, leading to increased IgG catabolism in the endosome–lysosome system. Rozanolixizumab and efgartigimod are recently developed agents targeting FcRn to accelerate pathological IgG degradation in autoimmune disorders. In phase II trials, nearly half of the persistent/chronic ITP patients treated with rozanolixizumab or efgartigimod achieved a response (platelet counts ≥ 50 × 10^9^/L). Both agents were well tolerated, with only mild-to-moderate adverse events [155, 156]. Phase III trials of rozanolixizumab or efgartigimod for ITP management are under way.

Sutimlimab, a humanized monoclonal antibody targeting C1s, has been tested for the management of patients with chronic/refractory ITP in a phase I trial [157]. Five of the 12 patients (42%) responded to biweekly intravenous infusion of sutimlimab (6.5 g or 7.5 g), and 4 (33.3%) achieved CR. A response could be maintained in every CR patient by prolonged sutimlimab administration. The median TTR was 2 days. Sutimlimab was generally well tolerated and no patients discontinued treatment due to drug-related adverse events.

Decitabine is a hypomethylating agent used for the treatment of MDS. Our previous studies indicated that low-dose decitabine could promote platelet production, revive Treg function, and inhibit the cytotoxic activity of CTLs toward platelets in ITP [158, 159]. In a prospective multicenter study, we found that low-dose decitabine treatment (3.5 mg/m^2^/day for 3 days) was well tolerated and could induce a response rate of 51% in refractory ITP patients, with a median TTR of 28 days. The SRRs at 6, 12, and 18 months were 44.4%, 31.1%, and 20%, respectively [160].

### Other agents

Many other immunosuppressive or immunomodulatory agents, such as azathioprine, cyclosporine A, danazol, dapsone, hydroxychloroquine, MMF, and vinca alkaloids, are also used in treating ITP. Evidence supporting their use is generally obtained from small retrospective studies. Depending on the population and agents used, approximately 30–60% of the ITP patients respond. Considering the emergence of new medications with promising efficacy, these immunosuppressive agents are used less frequently and are usually prescribed as rescue therapies for patients who fail multiple treatment options. A brief summary of treatments for ITP is presented in Table 2.

**Table 2:** Efficacy and safety of different treatments for ITP

Medications	Dosage	Time to response	Overall response	Response durability	Side effects
*Initial treatment*					
Prednisone	1 mg/kg/day for 1–2 weeks, then gradually tapper and stop by 6–8 weeks, rapid tapering in nonresponders	1–2 weeks	60–80%	30–50% of patients maintain the response after discontinuation	Weight gain, Cushingoid appearance, mood disorders, gastrointestinal toxicities, hyperglycemia, insomnia, hypertension, increased risks of infection, neuropsychiatric symptoms
HD-DXM	40 mg/day × 4 days, up to 2–4 cycles	1–9 days	60–80%	30–50% of patients maintain the response after discontinuation	Weight gain, gastrointestinal toxicities, hyperglycaemia, insomnia, hypertension, increased risk of infection, neuropsychiatric symptoms
IVIg	0.4 g/kg/day × 5 days or 1 g/kg/day × 1–2 days	1–4 days	Up to 80%	Transient	Headache, renal insufficiency, aseptic meningitis, anaphylactic reactions in IgA deficient patient
Anti-RhD	50–75 μg/kg	4–5 days	Up to 80%	Transient	Hemolysis, fever, chills, renal failure
*Subsequent treatment with robust evidence*					
Eltrombopag	25–75 mg/day	2–4 weeks	60–80%	40–60% with maintenance therapy, 10–30% keep the response after drug discontinuation	Headache, upper respiratory tract infection, diarrhea, hepatotoxicity, thromboembolic events, bone marrow fibrosis
Avatrombopag	5–40 mg/day	1–2 weeks	65% by day 8, 85% by day 28	70–80% with maintenance therapy	Headache, arthralgia, increased risk of thrombosis and bone marrow fibrosis
Hetrombopag	2.5–7.5 mg/day	15–25 days	58.9–64.3%	40–50% with maintenance therapy	Headache, upper respiratory tract infection, diarrhea, hepatotoxicity, increased risk of thrombosis and bone marrow fibrosis
Romiplostim	1–10 μg/kg once weekly	1–2 weeks	74–88%	40–60% with maintenance therapy	Headache, muscle aches, thrombosis, bone marrow fibrosis or increased reticulin
rhTPO	300 U/kg/day	1–2 weeks	60–75%	–	Drowsiness, dizziness, hypertension, fatigue, rash, urticaria, diarrhea
Rituximab	375 mg/m^2^ weekly × 4 weeks, 100 mg weekly × 4 weeks, 1000 mg on day 1 and day 15	1–8 weeks	60–80%	40–50%	Infusion-related reactions, hypogammaglobulinemia, increased risk of infections and HBV reactivation, progressive multifocal leukoencephalopathy
Fostamatinib	50–150 mg twice daily	1–2 weeks	40–50%	18–43% with maintenance therapy	Diarrhea, hypertension, nausea, dizziness, transaminitis
*Subsequent treatment with less robust evidence*					
Azathioprine	1–2 mg/kg/day	6–12 weeks	30–60%	Up to a quarter of patients off therapy keep the response after long treatment duration	Fatigue, transaminitis, neutropenia, increased risk of malignancy
Cyclosporin A	2.5–3 mg/kg/day, titration to blood levels of 100–200 ng/ml	3–4 weeks	50–80%	50% keep the response on low-dose maintenance	Hypertension, renal dysfunction, hypertrichosis, gingival hyperplasia, tremor
Cyclophosphamide	1–2 mg/kg/day orally for at least 16 weeks or IV 0.3–1 g/m^2^ 1–3 doses every 2–4 weeks	1–16 weeks	24–85%	Approximate 50%	Nausea, vomiting, hematuria, neutropenia
Danazol	200 mg, 2–4 times daily	3–6 months	30–60%	–	Acne, hirsutism, hypercholesterolemia, amenorrhea, transaminitis
Dapsone	75–100 mg/day	3–4 weeks	30–60%	–	Nausea, rash, dyspepsia methemoglobinemia, hemolytic anemia in glucose-6-phosphate dehydrogenase
MMF	1.5–2 g/day	4–8 weeks	30–60%	40%	Headache, gastrointestinal symptoms, increased risks of infection and cancer,
Vinca alkaloids	Vincristine: 1–2 mg weekly for 2–3 weeks Vinblastine: 10 mg weekly for 2–3 weeks	5–7 days	Transient. response in 10–75% of patients	–	Neuropathy, neutropenia, constipation, hepatotoxicity
*Novel agents under investigation*					
Rilzabrutinib	400 mg twice daily	1–10 weeks (median 11.5 days)	40%	–	Diarrhea, nausea, fatigue, infection
Rozanolixizumab	Starting at 4 mg/kg weekly, can be uptitrated to a maximum of 20 mg/kg weekly	1–2 weeks	35–45%	–	Headache, diarrhea, vomiting, pyrexia, infection
Efgartigimod	5 mg/kg or 10 mg/kg weekly × 4 weeks	8–43 days	38.5%	–	Rash, hypertension, vomiting, cystitis
Decitabine	3.5 mg/m^2^/day × 3 days for 3 cycles with a 4-week interval between cycles	2–10 weeks	51%	SR rate at 6 months was 44%	Nausea, fever, diarrhea, constipation, transaminitis
*Surgical treatment*					
Splenectomy	Open or laparoscopic surgery	immediately	75–90%	50–70%	Bleeding, infection, thrombosis, increased risk of cancer

*HD-DXM* high-dose dexamethasone, *IVIg* intravenous immunoglobulin, *rhTPO* recombinant human thrombopoietin, *MMF* mycophenolate mofetil

### Combination therapies

Although great progress has been made in ITP management during the last decade, there are still unmet needs with regard to the short- and long-term efficacy in many relapsed or refractory cases. A multicenter retrospective cohort study indicated that patients who failed splenectomy, rituximab, romiplostim, and eltrombopag had significantly increased morbidity and mortality, and the combination of immunosuppressants with TPO-RAs might be an effective strategy for these multirefractory cases [161]. Additionally, combination strategies have also been explored in prospective RCTs. Zhou et al. compared the efficacy of rituximab (100 mg/week for 4 weeks) plus rhTPO (300 U/kg/day for 14 days) with rituximab alone (100 mg/week for 4 weeks) in corticosteroid-resistant or relapsed ITP patients. The results showed that rituximab plus rhTPO had a significantly shorter TTR (7 days vs. 28 days) and remarkably reduced bleeding (24% vs. 45%) in the first 2 months, but the combination did not show superior SRR [162]. A phase II RCT conducted also in China demonstrated that danazol (200 mg twice daily for 16 weeks) plus ATRA (10 mg twice daily for 16 weeks) could induce a better SRR at 12 months (62% vs. 25%) and shorter TTR (35 days vs. 49 days) compared with danazol monotherapy [163]. More recently, another treatment modality using a combination of rituximab (100 mg weekly for 6 weeks) and ATRA (20 mg/m^2^ daily for 12 weeks) also yielded favorable outcomes in corticosteroid-resistant or relapsed patients, with a significantly higher overall response rate (80% vs. 59%) and SRR (61% vs. 41%) than rituximab alone [164]. These 3 combination strategies were well tolerated, and the severity of treatment-related adverse events was mostly grade 1 or 2. Other combination strategies, such as dexamethasone and low-dose rituximab combined with cyclosporine A, thrombopoietic agents plus immunosuppressants, or triple therapy with thrombopoietic agents, immunosuppressants, and IVIg, have also been tested for the management of refractory ITP in single-arm observational studies with overall response rates of 30–70%, thereby warranting further validation in larger trials.

## Strategies for selecting subsequent treatment

Direct head-to-head RCTs comparing the subsequent treatments of ITP are lacking. Although several studies have compared the efficacy of different subsequent treatments using network meta-analysis, caution should be taken when interpreting their results because of the heterogeneity in included patients, treatment duration, and definition of response. The reported predictors of response to subsequent treatments are often inconsistent across different studies. Selection of subsequent treatments is primarily based on individualized patient factors, including age, lifestyle, comorbidities, concomitant medications, response to previous treatment, and patient preference and adherence. Treatment factors such as cost-effectiveness and medication availability also occupy an important position in treatment selection. Among the treatment options with the most robust evidence, the updated ASH guidelines made conditional recommendations on the selection of TPO-RAs, rituximab, and splenectomy for patients under post-relapse conditions [12]. For the dichotomous evaluation of these 3 treatments, TPO-RAs were suggested over rituximab and rituximab over splenectomy; by contrast, one was not suggested over the other between TPO-RAs and splenectomy. It is notable that the updated ASH guidelines put a high value on patient preference in treatment decision-making; thus, patient education and shared decision-making are critical.

Our anecdotal experiences under several specific situations are listed as follows:

- In ANA-positive young female patients (aged < 40 years), rituximab is the preferred treatment. Hydroxychloroquine, an inexpensive and widely available agent commonly prescribed to treat SLE, can also be used in ANA-positive ITP patients, with a reported overall response rate of 50% [165].
- In postmenopausal female cases, we have a preference for danazol plus ATRA.
- In elderly patients with clonal hematopoiesis, we often prescribe low-dose decitabine as the first subsequent treatment.
- Thrombopoietic agents and splenectomy should be used with caution in cases at high risk of thromboembolism or with a history of thrombosis.
- Rituximab, immunosuppressive agents, and splenectomy should be avoided in cases with a history of recurrent infection or neoplasia.

## Special scenarios in ITP management

### ITP in pregnancy

Diagnosis of ITP in pregnancy is also based on the exclusion of other thrombocytopenic disorders. The differential diagnoses include gestational thrombocytopenia, pregnancy-related hypertensive diseases such as preeclampsia and HELLP syndrome, and other non-pregnancy-specific thrombocytopenic disorders. The recommended screening tests include complete blood counts, blood smears, reticulocyte counts, liver function, coagulation, thyroid function, ANAs, and APLAs. Pregnant ITP patients with a platelet count ≥ 20–30 × 10^9^/L and no bleeding symptoms can be managed with close observation only till close to term. A platelet count ≥ 50 × 10^9^/L is needed for normal delivery and ≥ 80 × 10^9^/L for safe spinal or epidural anesthesia. There are fewer treatment options for pregnant ITP patients compared to non-pregnant patients. Prednisone at 20 mg/day can be given initially and then tapered to the minimum dose necessary. IVIg infusion (1–2 g/kg) is useful in pregnant patients with an urgent need to increase platelet count. Patients who fail a single initial treatment can receive a combination of high-dose methylpredniso(lo)ne and IVIg. Moreover, rhTPO cannot cross the placenta and is appropriate for refractory cases [166]. Azathioprine and cyclosporine A can also be used as the subsequent treatments in pregnant patients with ITP. Other TPO-RAs and rituximab are not recommended in pregnant patients, although their off-label uses have been reported [167, 168]. Splenectomy is not considered an appropriate therapy for ITP in pregnancy. If necessary, splenectomy is best performed laparoscopically in the second trimester. Vinca alkaloids and MMF are forbidden during pregnancy.

### ITP and the COVID-19 pandemic

COVID-19 infection not only increases the incidence of ITP but also leads to the exacerbation of thrombocytopenia in previously diagnosed ITP patients [169, 170]. However, the mechanisms of thrombocytopenia after COVID-19 infection are usually multifactorial and quite different from the conventional model of platelet consumption in ITP. According to a recent meta-analysis, ITP secondary to COVID-19 usually occurs 2–3 weeks after severe acute respiratory syndrome coronavirus 2 (SARS-CoV-2) infection and recovers within 1 week [170], suggesting a different pathophysiological process compared to classical ITP. High-quality evidence about the optimal treatment for ITP patients during the COVID-19 pandemic is scarce, and recommendations from recently issued guidelines are primarily based on available case series and expert opinions [171, 172]. In general, TPO-RAs are preferred as the initial treatment in new/relapsed ITP patients who have not been infected with SARS-CoV-2, while prednisone at a low starting dose (usually 20 mg/day) and short duration is recommended for cases who are positive for COVID-19. IVIg (1 g/kg) is usually used in patients with an urgent need to elevate platelet counts, or as rescue therapy for patients who fail corticosteroids. If possible, rituximab and immunosuppressant agents should be avoided in new or relapsed patients. For patients with chronic stable ITP, their current medications should be maintained. COVID-19 infection is associated with a high risk of thrombotic complications [173, 174], and the infected ITP patients should be evaluated regularly for both bleeding and thrombotic risks throughout the disease course. Patients with a platelet count ≥ 30 × 10^9^/L and increased thrombotic risk should receive proper thromboprophylaxis, whether hospitalized or not. With regard to COVID-19 vaccination, several studies have supported the safety of the currently available vaccines in ITP patients, although approximately 10% of them might undergo clinical exacerbation [175, 176].

## Conclusions

ITP is a complex and heterogeneous disorder with uncertain etiology and ill-defined pathophysiology. Thrombocytopenia is the result of both increased platelet destruction and decreased platelet production, which is related to multiple abnormalities of the immune system in ITP. Diagnosis still relies on the exclusion of other thrombocytopenic diseases due to the lack of reliable biomarkers or gold-standard diagnostic tests. Although corticosteroids and IVIg remain the standard initial treatments for ITP, new explorations on the upfront use of agents such as TPO-RAs, MMF, and rituximab have depicted a landscape for future frontline therapies. Moreover, the emergence of novel agents targeting different pathogenetic mechanisms of ITP has deeply modified the second-line treatment modalities, which have gradually shifted away from immune suppression. This is exactly the situation during the COVID-19 pandemic. Patients’ HRQoL can also benefit from the use of non-immunosuppressive or less immunosuppressive agents. Patient preferences determined by treatment efficacy and potential complications take center stage in selecting the treatment regimen. However, there are still unmet needs in ITP management, such as implementing precise individualized treatment, avoiding overtreatment, and handling multirefractory cases. Therefore, the development of a stratification model capable of identifying patients who may truly benefit from treatment and guiding treatment selection is the priority research area. It would be better to incorporate the available clinical characteristics, immune profile, and environmental and genetic predispositions into the model, and validate it in future studies. Furthermore, precise shared decision-making tools should also be developed to optimize patient-specific treatment.

## Data Availability

Not applicable.

## Abbreviations

ITP: Primary immune thrombocytopenia
IVIg: Intravenous immunoglobulin
HRQoL: Health-related quality of life
IWG: International working group
ASH: American Society of Hematology
GP: Glycoprotein
FcγR: Fcγ receptor
CDC: Complement-dependent cytotoxicity
DC: Dendritic cell
IDO: Indoleamine 2,3-dioxygenase
Treg: Regulatory T cell
Th: T helper
HLA: Human leukocyte antigen (HLA)
AICD: Activation-induced cell death
TFH: Follicular Th
Breg: Regulatory B cell
CTL: Cytotoxic T lymphocyte
MGL: Macrophage galactose lectin
KCR: Kupffer cell receptor
CLEC4F: C-type lectin domain family 4
GalNAc: N-acetylgalactosamine
Ig: Immunoglobulin
ANA: Antinuclear antibody
APLA: Anti-phospholipid antibody
AA: Aplastic anemia
MDS: Myelodysplastic syndrome
MAIPA: Monoclonal antibody-specific immobilization of platelet antigen
DAT: Direct antiglobulin test
DNA: Deoxyribonucleic acid
WES: Whole-exome sequencing
SLE: Systemic lupus erythematosus
ITP-BAT: ITP-specific bleeding assessment tool
ICH: Intracranial hemorrhage
CMB: Cerebral microbleed
HD-DXM: High-dose dexamethasone
SRR: Sustained response rate
RCT: Randomized clinical trial
DIC: Disseminated intravascular coagulation
rhTPO: Recombinant human thrombopoietin
TPO-RA: Thrombopoietin receptor agonist
MMF: Mycophenolate mofetil
ATRA: All-trans retinoic acid
CR: Complete response
FDA: Food and Drug Administration
EMA: European Medicines Agency
RR: Risk ratio
TTR: Time to response
Syk: Spleen tyrosine kinase
BCR: B cell receptor
VTE: Venous thromboembolism
BTK: Bruton’s tyrosine kinase
FcRn: Neonatal Fc receptor
SARS-CoV-2: Severe acute respiratory syndrome coronavirus 2
